# Estimation of the UV susceptibility of aerosolized SARS-CoV-2 to 254 nm irradiation using CFD-based room disinfection simulations

**DOI:** 10.1038/s41598-024-63472-3

**Published:** 2024-07-10

**Authors:** Marc van der Schans, Joan Yu, Adrie de Vries, Genevieve Martin

**Affiliations:** Signify, High Tech Campus 7, 5656AE Eindhoven, The Netherlands

**Keywords:** Engineering, Fluid dynamics

## Abstract

The recent COVID-19 pandemic has raised interest in efficient air disinfection solutions. The application of germicidal ultraviolet (GUV) irradiation is an excellent contender to prevent airborne transmission of COVID-19, as well as other existing and future infectious airborne diseases. While GUV has already been proven effective in inactivating SARS-CoV-2, quantitative data on UV susceptibility and dose requirements, needed to predict and optimize the performance of GUV solutions, is still limited. In this study, the UV susceptibility of aerosolized SARS-CoV-2 to 254 nm ultraviolet (UV) irradiation is investigated. This is done by employing 3D computational fluid dynamics based simulations of SARS-CoV-2 inactivation in a test chamber equipped with an upper-room UV-C luminaire and comparing the results to previously published measurements performed in the same test chamber. The UV susceptibility found in this study is (0.6 ± 0.2) m^2^/J, which is equivalent to a *D*_90_ dose between 3 and 6 J/m^2^. These values are in the same range as previous estimations based on other corona viruses and inactivation data reported in literature.

## Introduction

Since the initial outbreak of the coronavirus disease 2019 (COVID-19) pandemic, more than 676 million cases and over 6.8 million casualties have been reported^1^. The virus responsible for the disease, SARS-CoV-2, spreads through respiratory particles produced by infected persons. While initially the virus was considered to be mainly transmitted through larger droplets, now airborne transmission through smaller aerosols containing SARS-CoV-2 is widely recognized as the principal mechanism by which the virus spreads^2–5^. As a results, there is an increased interest in air disinfection, particularly for indoor spaces. One method to achieve air disinfection is by safely using ultraviolet (UV) irradiation, which was recently considered as essential for airborne infection control in the future^4^.

UV technology has been applied to combat transmission of airborne infectious diseases for decades and is proven effective^6^. By now it has also been demonstrated in numerous studies that UV irradiation is capable of rapidly inactivating SARS-CoV-2 on surfaces, in liquid and in air^7–27^. Nevertheless, quantitative data on the dynamics and sensitivity of aerosolized SARS-CoV-2 to UV irradiation is still limited. To be able to predict and optimize the disinfection performance of GUV solutions, knowledge on UV susceptibility of SARS-CoV-2 and dose requirements is indispensable.

Generally the UV susceptibility of a pathogen depends on the wavelength of the UV light as well as the medium or state of the pathogen, i.e. whether the pathogen is aerosolized, suspended in a liquid, or on a substrate^28,29^. Light within the UV-C range of the spectrum, with wavelengths from about 200 nm to 280 nm, is particularly effective in inactivating pathogens. Low pressure Hg discharge lamps, which have strong line emission at 254 nm, are commonly used to produce UV-C light. Additionally, UV-C light-emitting diode (LED) technology has also emerged in UV disinfection research and applications in recent years^30,31^. In the context of aerosolized SARS-CoV-2, Beggs and Avital^29^ put forward a first prediction for the UV susceptibility at 254 nm*—*between 0.377 and 0.590 m^2^/J*—*based on data and knowledge of other (corona) viruses. To our knowledge, the only experimental inactivation data specifically for aerosolized SARS-CoV-2 exposed to 254 nm irradiation so far was reported by Ruetalo et al.^16^. They found that an exposure to 4.2–5.1 J/m^2^ of 254 nm irradiation resulted in more than 99.9% inactivation of aerosolized SARS-CoV-2. Although they suggest further experiments are needed to establish a firm dose–response curve, this measurement would imply a UV susceptibility of > 1.3 m^2^/J. Only a few other studies on the sensitivity of aerosolized SARS-CoV-2 to different UV-C wavelengths (222 nm, 265 nm, and 280 nm) have been published^17,18,20,32,33^. For the relatively close wavelength of 265 nm, Ueki et al.^18^ reported a reduction of 90%, 99% and 99.9% for doses of 2.3 J/m^2^, 4.0 J/m^2^ and 10.4 J/m^2^ respectively. This would correspond to a UV susceptibility between about 0.7 and 1.2 m^2^/J.

The goal of this study is to verify the predicted UV susceptibility range for aerosolized SARS-CoV-2 under 254 nm exposure. We do this by comparing measurements and simulations of the inactivation of aerosolized SARS-CoV-2 in a test chamber equipped with an upper-room 254 nm UV-C luminaire. The modelling approach we use in this study combines 3D computational fluid dynamics (CFD), particle tracking, and optical simulation of the UV-C radiation. The model is first tested and validated against the studies of Xu et al.^34^ on UV air disinfection. The model is then used in combination with experimental data from the study by Innovative Bioanalysis^35^ to estimate the UV susceptibility of aerosolized SARS-CoV-2 to 254 nm irradiation.

## Methods and materials

### Modelling approach

**Figure 1:**
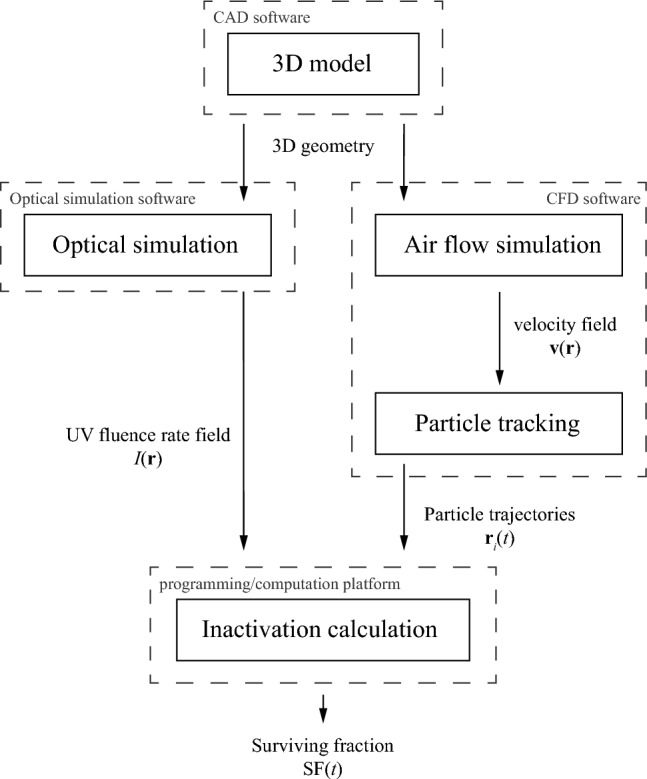
Schematic overview of the modelling approach used in this study.

Several approaches to aerosol transport and UV air disinfection modelling in a wide variety of conditions have been explored and reported in the past decades (see e.g.^36–47^). They range from relatively simple analytical models based on the assumption of complete mixing, to highly sophisticated CFD-based models. Analytical models based on complete mixing are useful for quick estimates and comparison between large numbers of different scenarios but, depending on the validity of the assumptions, may not always provide accurate results. On the other hand, high fidelity CFD models are able to capture far more detail, such as geometry and air flow patterns in the room, but are computationally demanding and time consuming. The modelling strategy that we use in this study is shown schematically in Fig. 1.

First a 3D model of the room of interest is made using commercially available computer-aided design (CAD) software (SolidWorks). The 3D geometry is transferred to both the optical simulation software and the CFD software.

An optical simulation is performed using a dedicated UV germicidal irradiation (UVGI) version of a commercially available tool (ReluxDesktop) to determine the UV fluence rate $I(\mathbf{r})$ (also called UV spherical irradiance) at coordinates $\mathbf{r}$ throughout the room. A photometric file describing the radiant intensity as a function of angle of the UV light produced by a UV-C luminaire can be used as input and is assigned to the location of the luminaire geometry in the 3D model. The 3D UV fluence rate data is exported from the software for further processing in the inactivation calculation step.

The air flow in the room is calculated using commercially available CFD software (Siemens Simcenter FLOEFD) under steady-state conditions using a Reynolds-averaged Navier–Stokes (RANS) turbulence model. The software uses an implementation of the *k*–ε model^48^. While transient simulations and/or more intricate turbulence models, such as large eddy simulation (LES), could potentially predict indoor air flow with greater accuracy^49^, the current approach was chosen to limit computational expense.

Once the velocity vector field $\mathbf{v}(\mathbf{r})$ is obtained from the air flow simulation, Lagrangian particle tracking is employed to quantify the transport of aerosols in the room over time. A sufficiently large number ${N}_{0}$ of particles, to be further specified later, is released in a manner that matches the aerosol generation in the experiments. For each of the individual particles *i*, the coordinates ${\mathbf{r}}_{i}(t)$ at time $t$ are recorded as it traverses the room. Lagrangian particle tracking assumes that the motion of the particles follows and does not influence the bulk fluid flow. Previous studies on aerosol transport have indicated that aerosols indeed follow the bulk fluid and act as tracers^50,51^. Particle collisions, mergers, and depositions are not considered in our model, although the latter will be implicitly accounted for in the inactivation calculation step. Furthermore, it is assumed that no heat or mass transfer takes place between the air and the particles.

In the inactivation calculation, we use the UV fluence rate data and particle trajectory data to calculate the number of active particles that are left in the room after time $t$, relative to the initial number of particles ${N}_{0}$ at $t=0$. Several mechanisms contribute to the decrease of active particles in the room. Here we will make a distinction between contributions attributed to ventilation, the germicidal action of the UV irradiation, and the collective contribution of other processes, such as aerosol deposition and natural decay of the pathogen.

When ventilation is active, particles will eventually be extracted from the room. From the particle trajectories the residence time ${\tau }_{i}$ is determined for each particle $i$. Whether particle $i$ is still present in the room (${p}_{i}=1$) or has been extracted (${p}_{i}=0$) at time $t$ can mathematically be described as

1 $$p_{i} \left( t \right) = 1 - H\left( {t - \tau_{i} } \right),$$

where $H\left(t\right)$ is the Heaviside step function. The number of particles still in the room ${N}_{\text{room}}\left(t\right)$ at time $t$, active or otherwise, is then

2 $$N_{{{\text{room}}}} \left( t \right) = \mathop \sum \limits_{i} p_{i} \left( t \right).$$

As the particles traverse the room, they are exposed to the UV radiation. The cumulative UV dose ${D}_{i}\left(t\right)$ acquired by particle $i$ at time $t$ is given by

3 $$D_{i} \left( t \right) = \mathop \smallint \limits_{0}^{t} I_{i} \left( {t^{\prime}} \right){\text{d}}t^{\prime}$$

where ${I}_{i}(t)$ is obtained by evaluating the UV fluence rate field $I(\mathbf{r})$ at the trajectory coordinates of particle *i* as

4 $$I_{i} \left( t \right) = I\left( {{\mathbf{r}}_{i} \left( t \right)} \right).$$

Assuming a single stage decay process, the fraction of pathogens ${f}_{i}(t)$ traveling along particle trajectory $i$ and still active after being exposed to a UV dose ${D}_{i}\left(t\right)$ can be described by

5 $$f_{i} \left( t \right) = \exp \left( { - \left[ {kD_{i} \left( t \right) + \lambda_{{{\text{eff}}}} t} \right]} \right),$$

where $k$ is the pathogen’s UV susceptibility and ${\lambda }_{\text{eff}}$ is the effective natural decay constant^28,36,52^.

The number of particles $N\left(t\right)$ that are statistically still active and in the room at time $t$ is obtained from (1) and (5) as

6 $$N\left( t \right) = \mathop \sum \limits_{i} p_{i} \left( t \right)f_{i} \left( t \right).$$

The surviving fraction $\text{SF}(t)$ of pathogens, relative to the initial number ${N}_{0}$, is then

7 $${\text{SF}}\left( t \right) = \frac{N\left( t \right)}{{N_{0} }}.$$

Calculated surviving fractions can be compared directly against measured values. To assess and compare the performance of a GUV system, metrics that can be derived from $\text{SF}(t)$ are often used. When $\text{SF}(t)$ follows an exponential decay curve, a common approach is to fit an exponential decay model of the form $\text{exp}(-\lambda t)$ to $\text{SF}(t)$^28^. The obtained decay constant $\lambda$ (in s^−1^) can subsequently be used to determine the so-called equivalent air changes per hour (eACH, in h^−1^) as $\text{eACH}=3600$ λ.

The inactivation calculation as described above is implemented using the Python programming language. The fluence rate is calculated on a 3D rectilinear grid. The fluence rate values at the trajectory coordinates in (4) are calculated using linear interpolation. The integration in (3) is performed numerically using the composite trapezoidal rule.

Our approach to modelling the effects of UV disinfection is somewhat similar to the one used in^43^, which also uses particle tracking. Alternatively, a passive scalar field approach^40–42,44,45,47,53,54^ could have been used instead of particle tracking. In this case an additional equation for the aerosol or pathogen number density (i.e. the concentration) is solved in the CFD software. The advantage is that the concentration is calculated throughout the entire room at once. On the other hand, an advantage of the approach presented here is that it is relatively straightforward to implement with any choice of software package for each of the steps, and no programming or dedicated UVGI functionality is needed in the CFD software.

### Verification and validation of the modelling approach

To verify and validate the modeling approach and to estimate the magnitude of several uncertainties and errors, a case reported in literature, for which the UV susceptibility is known, is simulated. The calculated surviving fractions are subsequently compared to experimental measurements. In particular, we use the UV inactivation study on *M*.* parafortuitum* ($k=0.12$ m^2^/J) performed and reported by Xu et al.^34^.

**Figure 2:**
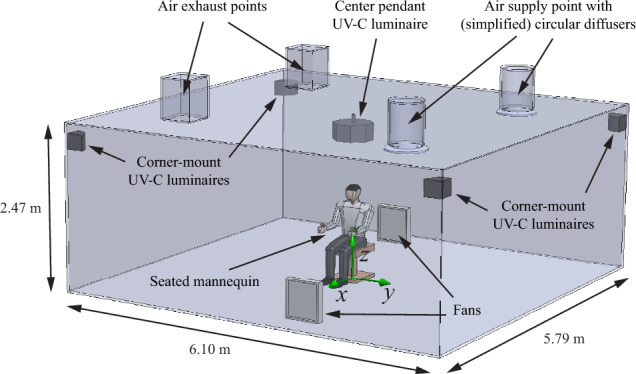
The 3D model used in the model verification and validation, based on the measurement facility of Xu et al.^34^.

The measurement facility used in the work of Xu et al. has been described and characterized in several publications^34,55–58^. A 3D model of the room, based on the aforementioned publications, is shown in Fig. 2. The room has a floor area of approximately 6.10 m by 5.79 m and is 2.47 m high. The mechanical ventilation system consists of two air supply points, each with a circular diffuser, and two air exhaust points, located on opposite sides of the room. In addition to the mechanical ventilation, two box fans (Lasko Inc. 3723) are placed on the floor to promote air mixing. To emulate an infected person producing pathogen-containing aerosols, a mannequin in seated position is situated in the middle of the room. The mannequin is covered in heating tape producing 108 W of heat. The room is equipped with five UV-C luminaires: one pendant UV-C luminaire (Lumalier PM) in the center of the room, and a corner-mount UV-C luminaire (Lumalier CM-218) in each of the four corners of the room. The top sides of the luminaires are located approximately 10 cm from the ceiling.

Experiments have been conducted by Xu et al. with the mechanical ventilation operating at 0, 3, and 6 ACH, and the UV-C luminaires operating at 100% (full power), 50% (half power), and 0% (off). The room temperature and relative humidity were kept at 24 °C and 50% respectively. Before the actual measurement is started, pathogen-containing aerosols are emitted in the region near the mannequin’s head, about 1 m from the floor, and allowed to disperse throughout the room. During this dispersion time the fans are already running, but the mechanical ventilation and UV-C system are still switched off. Then, at $t=0$, the generation of aerosols is stopped, the mechanical ventilation and UV-C system are switched on, and the measurement starts. Surviving fraction data have been reported for 16 min at intervals of 4 min.

Since we have no photometric files available for the luminaires used in the measurements, and the UV reflectivity of the ceiling and wall materials are also unknown, we cannot calculate the exact UV fluence rate distribution. Nevertheless, Xu et al.^34^ performed fluence rate measurements and report that the average height of the UV band in the upper room is 30 cm, and that the spatially averaged fluence rate in this band is 0.42 W/m^2^ when the UV-C luminaires operate at full power (100% UV) and 0.2 W/m^2^ when the UV-C luminaires operate at half power (50% UV). Based on this information we will consider two artificial fluence rate distributions to estimate the uncertainty this introduces in our modelling results. The first distribution will have a uniform fluence rate 0.42 W/m^2^ (or 0.2 W/m^2^ for 50% UV) in a 30 cm wide band in the upper room and 0 W/m^2^ everywhere else in the room. The second, nonuniform, distribution will treat the luminaires as ideal line sources, located in the center and each of the corners. The resulting fluence rate field will be scaled to match the average value of 0.42 W/m^2^ (or 0.2 W/m^2^ for 50% UV) in a 30 cm wide band in the upper room and set to 0 W/m^2^ in the rest of the room.

To mimic the operating conditions of the experiments in the air flow simulation, we set the wall temperature in our model to 24 °C and apply a heat source of 108 W to the surface of the mannequin. The mechanical ventilation is realized by imposing a volume flux on the air inlet point, calculated as the air changes per hour multiplied by the volume of the room. Additionally, a static pressure boundary condition of 1 atm is applied to the air exhaust points. The air flow produced by the two fans is implemented by imposing a volume flux on the side of the fan that faces into the room, and a negative volume flux of equal magnitude on the other side. Unfortunately, the setting of the fans in the experiments is unknown, but according to the manufacturer the minimum and maximum volume fluxes of these fans are about 0.75 m^3^/s (1600 cfm) and 0.95 m^3^/s (2000 cfm) respectively. We will use a volume flux of 0.85 m^3^/s as baseline in our model and include this parameter in the uncertainty analysis to see its influence on the results.

The heat generated by the luminaires can be accounted for in the model by applying heat sources to the luminaires. While the heat generation is likely close to the electrical rating of the luminaires (72 W for the pendant luminaire, and 36 W for each of the four corner-mount luminaires), the exact dissipation is unknown. The effect of the heat generated by the UV-C luminaires will also be considered as a contributor to the uncertainty in the results.

To match the initial locations of the particles at $t=0$, the particles are released directly in front of the face of the mannequin and allowed to disperse for 14 min throughout the room in the air flow corresponding to the 0 ACH scenario, i.e. with the mechanical ventilation turned off. Whenever a particle enters one of the fans, it is ejected at a random location on the room-facing surface of the same fan to emulate the motion of the particle inside the fan. The locations of the particles after these 14 min are subsequently used as the initial positions of the particles that are tracked and used in further analysis for each of the cases.

The (geometric) mean aerosol diameter in the experiments is reported as 1.6 ± 1.2 µm. Although it is in principle possible to use a particle size distribution in the model, we use a single particle diameter of 0.8 µm. Nevertheless, two other particles sizes, which together cover the entire size range, will also be tested, namely 0.1 µm (approximately the size of SARS-CoV-2^59^) and 5 µm (commonly considered the upper limit for airborne aerosols). In every case the particles are assigned the mass density of water.

Finally, besides a sufficiently dense computational mesh in the air flow simulation, our approach also requires a sufficiently large number of particles in the room at any time $t$ for convergence of the results. This condition is most stringent when the number of air changes per hour (ACH) by the mechanical ventilation is high, as particles are then more rapidly extracted from the room. To determine the number of particles needed for our study, and the corresponding error that can expected, we will compare results of the 6 ACH case obtained with a set of 100,000 initial particles and randomly sampled subsets with sample sizes of 10, 100, 1000 and 10,000 particles.

### Quantification of UV susceptibility

When the UV susceptibility of a pathogen is unknown, the model can be used together with an optimization routine to find the UV susceptibility value for which the simulated data fits best to experimental data. We use this strategy to determine the UV susceptibility of aerosolized SARS-CoV-2 to 254 nm irradiation using our model and experimental data reported by Innovative Bioanalysis^35^ as a commercial service.

**Figure 3:**
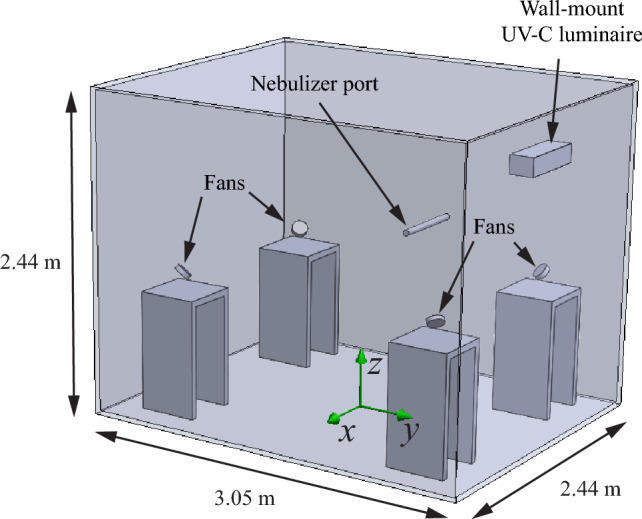
The 3D model used in the quantification of the UV susceptibility of aerosolized SARS-CoV-2 to 254 nm irradiation, based on the testing chamber of Innovative Bioanalysis^35^.

A 3D model of the testing chamber used in the work of Bioanalysis is shown in Fig. 3. The overall dimensions of the chamber are approximately 3.05 m by 2.44 m by 2.44 m. In contrast to the room of the verification and validation case, this testing chamber is air sealed and hence there is no ventilation. To ensure effective air mixing a fan is placed in each of the corners of the room on a pedestal that is about 91 cm high. The fans are pointed upward in a 45° angle, facing diagonally into the room, and operate at a volume flux of about 0.015 m^3^/s. A single 254 nm UV-C luminaire (Philips WL345W) is mounted in the middle of one of the side walls approximately 2.1 m above the floor. Aerosolized SARS-CoV-2 is introduced in the test chamber through the nebulizer port, which is located at the center of the back wall, at a height of approximately 1.72 m, and protrudes about 61 cm into the room.

The experiments of Innovative Bioanalysis were conducted at a temperature of approximately 20 °C and a relative humidity of 50%. The aerosols containing SARS-CoV-2 are allowed to disperse into the testing chamber with the fans in operation for about 10 min. Then, at $t=0$, 254 nm radiation is introduced into the testing chamber, by removing a shutter from the UV-C luminaire, and the measurement starts. Surviving fraction data has been reported at 2, 5, 10, and 20 min. However, the data point at 20 min was below the measurement threshold and will therefore not be included in our analysis.

A photometric file of the luminaire is used as input for the optical simulation. The light source is assigned to the center of the luminaire geometry. The surface reflectivity of the wall material of the test chamber was measured to be 6% for 254 nm light and it is set accordingly in the model.

In the air flow simulation, a temperate of 20 °C and a relative humidity of 50% are used to match the experimental conditions. The air flow produced by the fans is realized in the model by imposing volume fluxes of 0.015 m^3^/s and − 0.015 m^3^/s on the front surface and back surface of each fan respectively. The thermal dissipation of the luminaire is neglected in this case.

The particles used for particle tracking are released from the circular face of the nebulizer port. To emulate the initial dispersion throughout the room, the particle positions after 10 min are taken as starting points for the trajectories later used in the inactivation calculation. The aerosols are again modeled as spherical water particles with a single size diameter of 0.8 µm, which matches the average size reported by Innovative Bioanalysis^35^.

An optimizer is used in the inactivation calculation step to find the UV susceptibility value that produces results that best fit the measurement data. The objective function that is minimized by the optimization routine is the sum of squares of the differences between the measured and simulated surviving fractions. The value $k=0.5$ m^2^/J, which is about in the middle of the range predicted by Beggs and Avital^29^, is used as initial guess.

## Results and discussion

### Verification and validation results

Before comparing our modelling results to the experimental data of Xu et al. for validation, we first need to consider several other aspects of the simulations. In particular, we will first look at the initial distribution of particles in the room at $t=0$, the effects of the number of particles, and the uncertainties introduced by the unknown inputs. Additionally, we need to determine the effective natural decay constant ${\lambda }_{\text{eff}}$ from the 0 ACH and 0% UV data.

**Figure 4:**
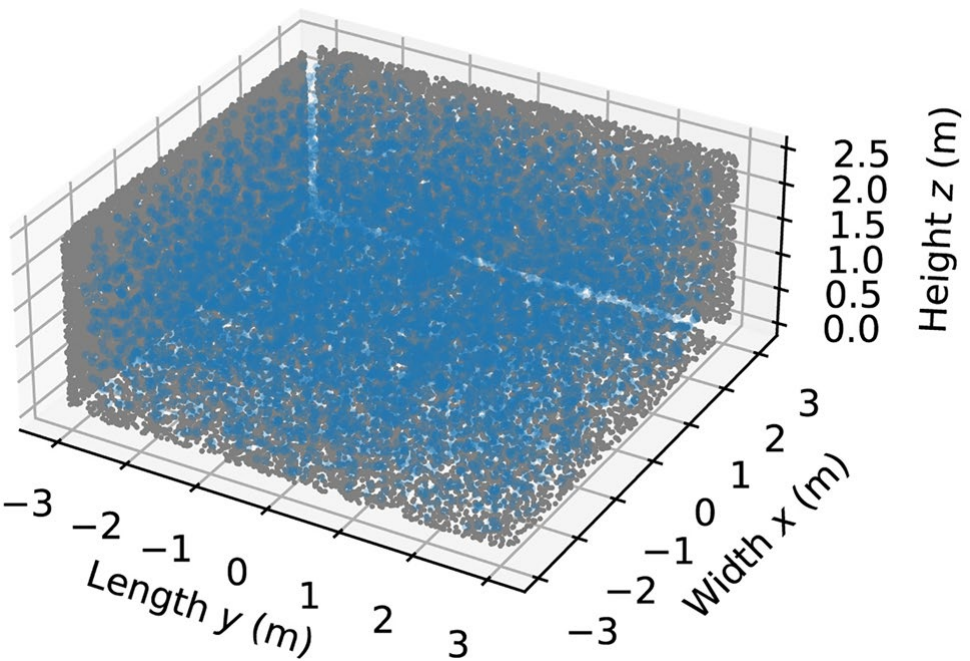
Initial distribution (at $t=0$) of the particles. Each of the blue markers indicates the 3D spatial coordinates of one of the particles. The grey markers show the particle coordinates projected onto the xy-, yz-, and xz-planes.

Figure 4 shows the initial distribution of particles in the room, after dispersion, at $t=0$. Although the particles are all released in vicinity of the mouth of the mannequin, they are dispersed nearly uniformly throughout the room at $t=0$. This is an indication that the air in the room is well mixed by the two fans, which was also observed experimentally using tracer gas measurements in^34,56^.

**Figure 5:**
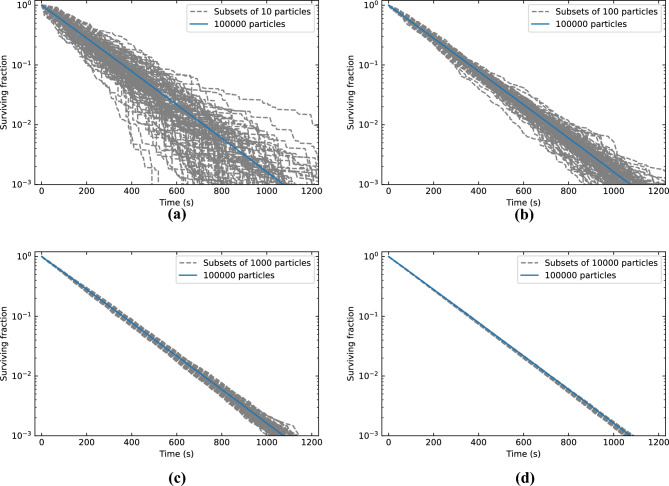
Effects of the initial particle number on the calculated surviving fraction. In each of the graphs the solid blue line indicates the results obtained using the full set of 100,000 particles. The dashed grey lines show the results obtained using randomly sampled subsets of (**a**) 10 particles, (**b**) 100 particles, (**c**) 1000 particles, and (**d**) 10,000 particles.

The effect of initial particle number is illustrated in Fig. 5. For each of the sample sizes, a hundred subsets were randomly sampled from the full set of 100,000 particles. The deviations in the subsets increase over time as the number of particles in the room decreases. This effect is most severe for lower initial particle numbers, as expected. The resulting relative error in the decay constant, calculated as the standard deviation of the different samples, are 23%, 9%, 3%, and 1% for 10, 100, 1000, and 10,000 initial particles respectively. Since a 1% relative error in the decay constant is deemed sufficiently accurate for the purpose of this study, all further simulations are performed with 10,000 particles.

**Figure 6:**
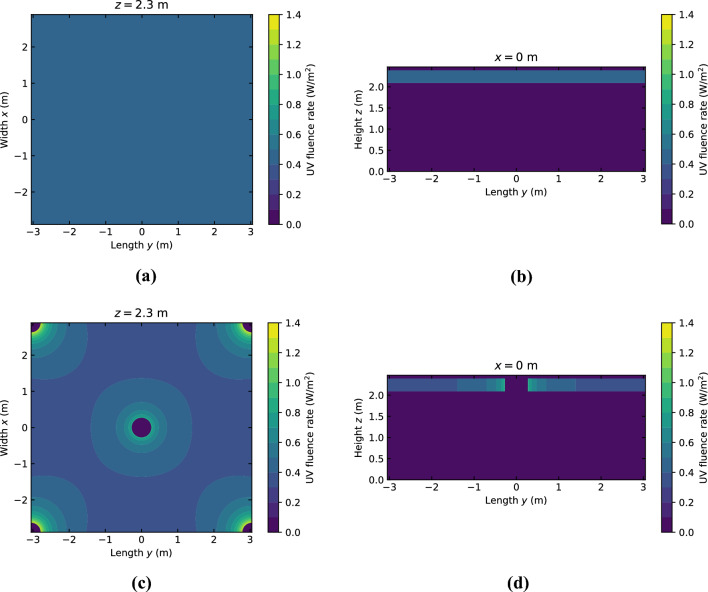
UV fluence rate distribution used in the analysis for the verification and validation case. In (**a**) and (**b**) a horizontal and vertical cross-section are shown respectively for the uniform distribution. In (**c**) and (**d**) a horizontal and vertical cross-section are shown respectively for the nonuniform distribution.

**Figure 7:**
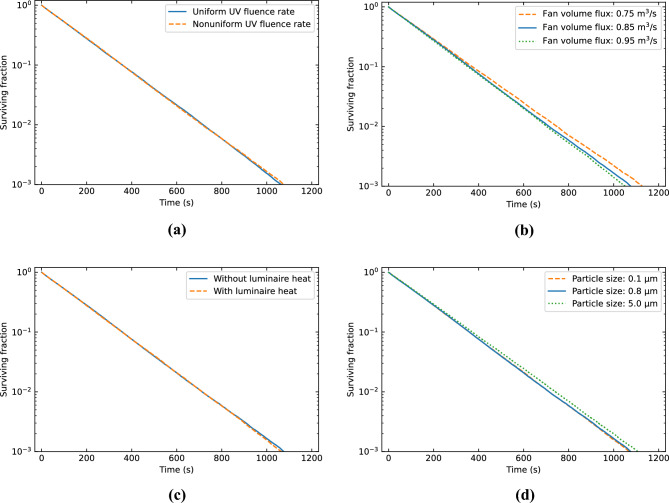
Effects of the (**a**) UV fluence rate distribution, (**b**) fan volume flux, (**c**) luminaire heat, and (**d**) particle size on the results of the verification and validation case.

The two UV fluence rate fields used to estimate the uncertainty in the results related to the fact that we do not know the exact distribution are shown in Fig. 6. In the situation where the UV-C luminaires are treated as ideal line sources, the UV fluence rate is highest close to each of the luminaires as expected. Nevertheless, the layout of the UV-C luminaires still provides a relatively uniform UV fluence rate in large parts of the upper room. The surviving fractions calculated with each of the two UV fluence rate fields are shown in Fig. 7a. Apart from small deviations, the results are nearly identical and the difference in decay constants obtained from this data is only about 1%. This is likely another indication that the air in the (upper) room is indeed well mixed in this case.

In Fig. 7b, the influence of the fan volume flux on the surviving fraction is depicted. Higher volume fluxes lead to increased decay rates, most likely due to improved air mixing. The uncertainty in the fans operating point introduces a 5% uncertainty in the decay constant obtained from the surviving fraction.

The effect of the heat produced by the UV-C luminaires is shown Fig. 7c. The difference in decay rate is less than 1%. Since a separate simulation, which included the luminaire heat, and particle set were used in the analysis, this difference could be due to the error between different particle sets that we found earlier. The effect of luminaire heat therefore appears negligible in this case. A plausible reason for this is that the air mixing in the room is completely dominated by the air flows induced by the fans and mechanical ventilation. It should be noted however that this may be different for cases where no forced air flows are present.

The surviving fractions obtained with different particle sizes are plotted in Fig. 7d. The decay rate is the practically the same for particles of size 0.1 µm and 0.8 µm, and slightly slower for particles of 5 µm. The difference in decay constants is about 3%. So, for the analysis of particle trajectories and aerosol transport in our study the actual particle size distribution is expected to have no major impact and introduces a small uncertainty in the results. While the aerosol size and composition may in practice influence the UV dose received by individual viruses and their inactivation process^60,61^, such effects are beyond the scope of our investigation.

**Figure 8:**
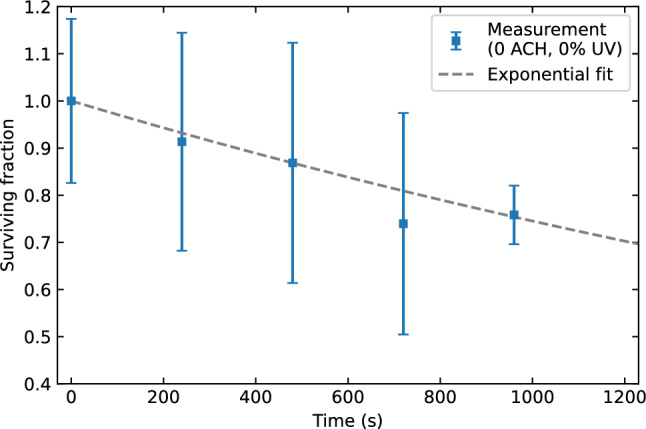
Measurement data of Xu et al.^34^ for the 0 ACH and 0% UV scenario (blue markers) and an exponential fit (grey dashed line) to determine the effective natural decay constant.

Now we will determine the effective natural decay constant ${\lambda }_{\text{eff}}$ for the verification and validation case using the 0 ACH and 0% UV scenario. In Fig. 8 the measurement data and exponential fit are shown. The effective natural decay constant is ${\lambda }_{\text{eff}}=\left(2.9\pm 0.8\right)\times {10}^{-4}$ s^−1^. This value for ${\lambda }_{\text{eff}}$ is assumed to be unaffected by the ventilation rate and will be used as input in the analysis of all ventilation scenarios, together with the earlier discussed 10% total estimated uncertainty.

**Figure 9:**
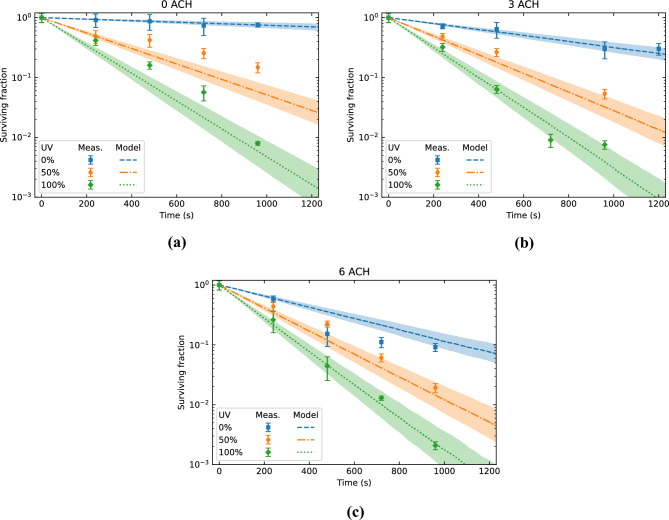
Comparison of the measurements of Xu et al.^34^ (markers) and the results of our model (lines) for (**a**) 0 ACH, (**b**) 3 ACH, and (**c**) 6 ACH. The shaded areas around the lines indicate the model’s estimated error and uncertainties related to the input parameters.

**Figure 10:**
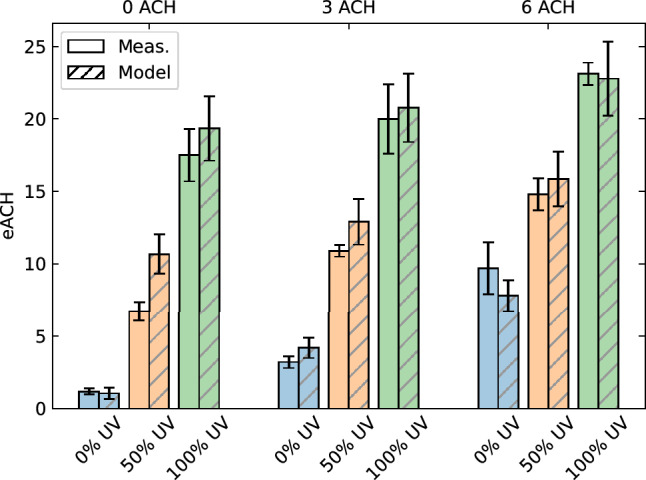
Comparison of the decay constants extracted from the measurements of Xu et al.^34^ and our model.

A comparison of the surviving fractions measured by Xu et al. and simulated by our model are shown in Fig. 9. We observe that our model matches the largest part of the individual experimental data points within the uncertainty levels. The extracted decay constants, expressed in eACH, are compared to those reported by Xu et al. in Fig. 10. The only significant difference between the experimental data and our model is found for the scenario at 0 ACH with 50% UV. However, since the results do match for the other 0 ACH scenarios as well as the other 50% UV scenarios, it is unclear what is responsible for this deviation. Although there is no direct evidence towards the cause, one possible explanation could be that the mixing conditions were different during that particular experiment. Nevertheless, overall, we conclude that our modelling approach produces realistic inactivation results with an uncertainty of about 10%.

### UV susceptibility of aerosolized SARS-CoV-2

**Figure 11:**
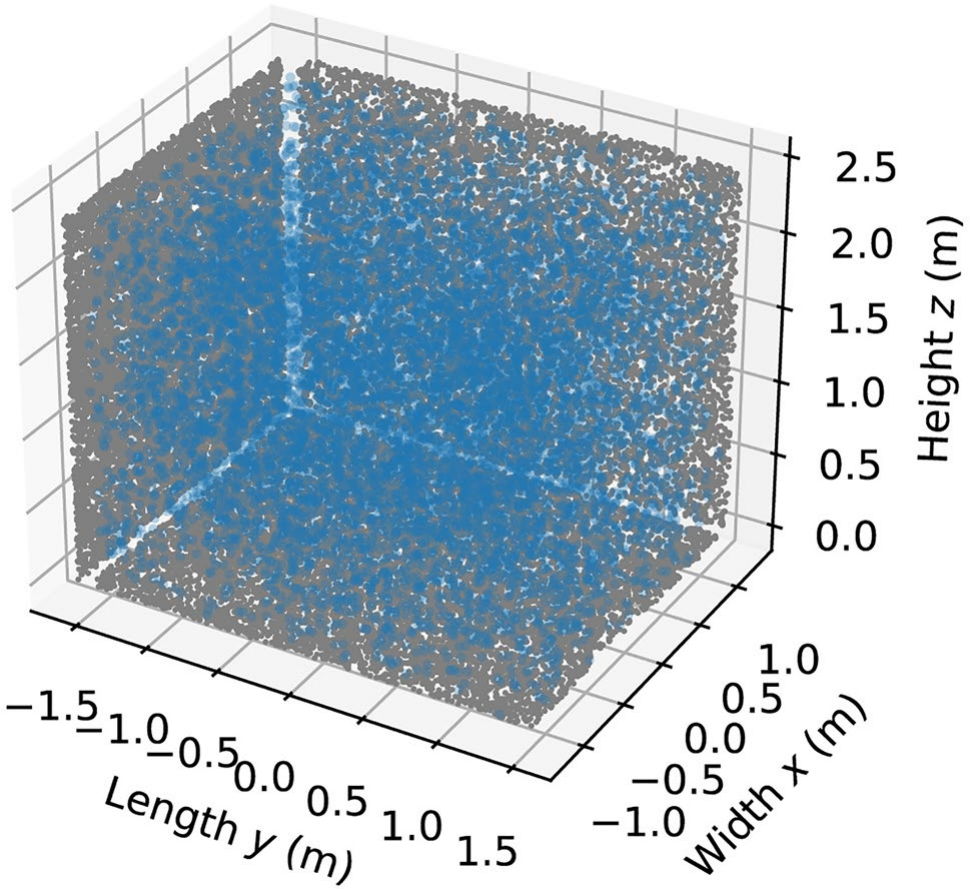
Initial distribution (at $t=0$) of the particles for the aerosolized SARS-CoV-2 case. Each of the blue markers indicates the 3D spatial coordinates of one of the particles. The grey markers show the particle coordinates projected onto the xy-, yz-, and xz-planes.

First, we will again consider the initial particle distribution and the UV fluence rate distribution in the test chamber. In Fig. 11 the locations of the particles at $t=0$ are illustrated. Like the previous case, the air flow provided by the four fans leads to a nearly uniform initial particle distribution. This indicates good air mixing conditions in the testing chamber.

**Figure 12:**
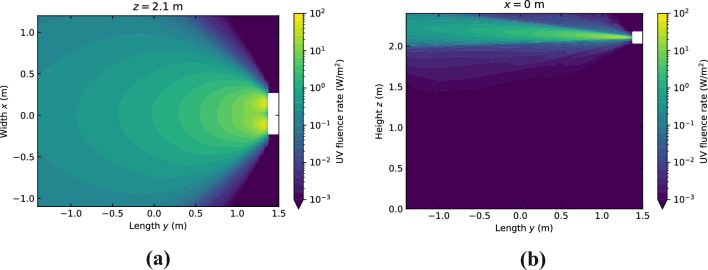
UV fluence rate in the testing chamber during the measurements on aerosolized SARS-CoV-2. In (**a**) and (**b**) a horizontal and vertical cross-section are shown respectively.

Two cross sections of the UV fluence rate field are shown in Fig. 12. The fluence rate values were exported from the optical simulation software on a 5 × 5 × 5 cm grid (length × width × height) in the lower part of the test chamber (*z* < 1.8 m) and a 5 × 5 × 1 cm grid in the upper part of the room (*z* > 1.8 m). By testing different sizes this was found to sufficiently resolve the fluence rate gradients in the test chamber. In the horizontal cross-section of Fig. 12a it appears as if the UV light source consists of two discrete point sources. This is an artefact related to the way the optical simulation software handles extended light sources. Therefore, the near field fluence rate close to the luminaire is not completely accurate. However, we found that this has no significant influence on the inactivation results. The average UV fluence rate over the entire testing chamber is approximately 0.05 W/m^2^.

**Figure 13:**
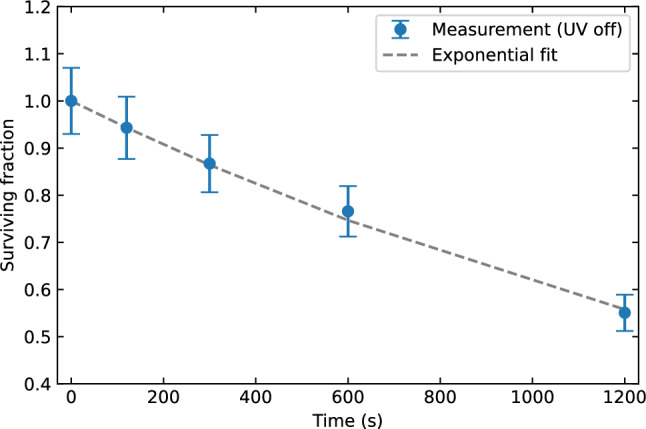
Control measurement data of Innovative Bioanalysis^35^ with the UV-C luminaire switched off (blue markers) and an exponential fit (grey dashed line) to determine the effective natural decay constant.

Before we can infer the UV susceptibility of aerosolized SARS-CoV-2 to 254 nm irradiation from the model and measurement data, we still need to determine the effective natural decay constant for this case. The control measurement data, for which the UV luminaire was switched off, is shown in Fig. 13 together with an exponential fit. A value of ${\lambda }_{\text{eff}}=\left(4.8\pm 0.7\right)\times {10}^{-4}$ s^−1^ is found. This value of effective natural decay constant is of the same order or magnitude as the one found in the verification and validation results. Differences in the exact value are to be expected, as the effective natural decay depends on the natural decay of the specific pathogen species as well as the rate at which aerosols deposit in the room.

**Figure 14:**
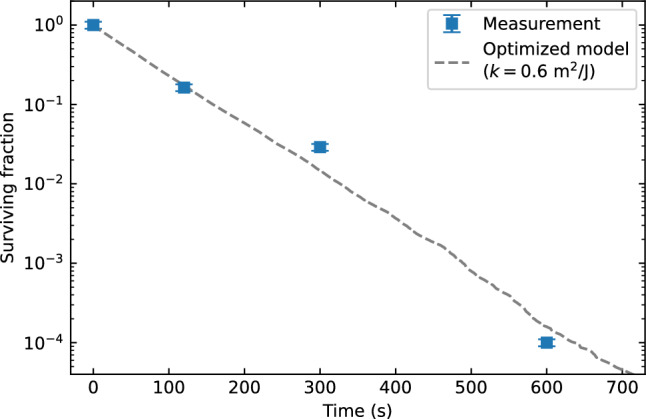
Comparison of the surviving fraction of aerosolized SARS-CoV-2 obtained from the measurements performed by Innovative Bioanalysis^35^, and from our model after optimizing the UV susceptibility to best match the measurements.

Finally, the results of the model with optimized UV susceptibility value are shown Fig. 14 together with experimental data of Innovative Bioanalysis. Based on the measurement data, the model predicts that the UV susceptibility of aerosolized SARS-CoV-2 to 254 nm irradiation is $k=0.6\pm 0.2$ m^2^/J. This corresponds to a ${D}_{90}$, i.e. the dose at which 90% of pathogens is inactivated, of about 3 to 6 J/m^2^.

Comparing the UV susceptibility found in this study ($0.6\pm 0.2$ m^2^/J) to the range predicted in literature based on other corona viruses (0.377–0.590 m^2^/J)^29^, and to the values derived from experimental work of Ruetalo et al.^16^ (> 1.3 m^2^/J) and Ueki et al. (0.7–1.2 m^2^/J)^18^, we find that they are all within the same order of magnitude. However, they are not mutually consistent. There are several factors that might contribute to these differences.

Firstly, validating the exact UV dose received by the pathogen-containing aerosols remains challenging, both in experiment and simulation. The air flow and the fluence rate distribution need to be precisely known and match the experimental conditions. Any deviations will directly impact the obtained UV susceptibility value. While the predicted inactivation rates produced by our simulation method were validated against a known case from literature, there is unfortunately no data available to individually validate the simulated air flow and fluence rates present in the testing chamber in which the experiments with aerosolized SARS-CoV-2 were performed. Additionally, our analysis would have benefitted from a larger number of experimental data points. Since the surviving fraction in the model is one at $t=0$ by definition, effectively only three data points could be used in the analysis.

The chosen experimental setup and conditions might also influence the obtained value for the UV susceptibility. As mentioned before, differences in relative humidity, aerosol size distribution and composition may influence the UV dose received by individual viruses and their inactivation process^28,60,61^. Furthermore, studies on multiple variants of SARS-CoV-2 have indicated differences in UV sensitivities between variants^62–64^.

More research is needed to further consolidate the UV susceptibility of aerosolized SARS-CoV-2 to 254 nm irradiation. Instead of using a relatively large testing chamber, we recommend that dose–response measurements are performed in a bench top setup, as previously suggested by Ruetalo et al.^16^, and as performed for 222 nm by Welch et al.^33^. In such a setup the air flow and fluence rate, and thereby the received UV dose, should be easier to control and validate than in a larger room, such as the one that was analyzed in the current study. Nevertheless, the currently available knowledge on the UV susceptibility range, together with a room disinfection modelling approach as described in this work, should provide a good starting point for the assessment and optimization of GUV solutions targeting aerosolized SARS-CoV-2 with 254 nm irradiation.

## Data Availability

The measurement data used in this study is available online (see^34,35^). Additional data supporting the findings of this study are available from the authors on reasonable request.
